# Dimethylformamide Impurities as Propylene Polymerization Inhibitor

**DOI:** 10.3390/polym15183806

**Published:** 2023-09-18

**Authors:** Joaquín Hernández-Fernández, Rafael González-Cuello, Rodrigo Ortega-Toro

**Affiliations:** 1 Chemistry Program, Department of Natural and Exact Sciences, San Pablo Campus, University of Cartagena, Cartagena 130015, Colombia; 2 Chemical Engineering Program, School of Engineering, Universidad Tecnológica de Bolivar, Parque Industrial y Tecnológico Carlos Vélez Pombo, Km 1 Vía Turbaco, Turbaco 130001, Colombia; 3 Department of Natural and Exact Science, Universidad de la Costa, Barranquilla 30300, Colombia; 4 Food Packaging and Shelf-Life Research Group (FP&SL), Food Engineering Program, Universidad de Cartagena, Avenida del Consulado St. 30, Cartagena de Indias 130015, Colombia; rgonzalezc1@unicartagena.edu.co (R.G.-C.); rortegap1@unicartagena.edu.co (R.O.-T.)

**Keywords:** polypropylene, N,N-dimethylformamide (DMF), Ziegler–Natta catalyst, productivity, melt flow index (MFI), molecular weight distribution (MW), catalyst inhibition, density functional theory (DFT)

## Abstract

This research study examined how the use of dimethylformamide (DMF) as an inhibitor affects the propylene polymerization process when using a Ziegler–Natta catalyst. Several experiments were carried out using TiCl_4_/MgCl_2_ as a catalyst, aluminum trialkyl as a cocatalyst, and different amounts of DMF. Then, we analyzed how DMF influences other aspects of the process, such as catalyst activity, molecular weight, and the number of branches in the polymer chains obtained, using experimental and computational methods. The results revealed that as the DMF/Ti ratio increases, the catalyst activity decreases. From a concentration of 5.11 ppm of DMF, a decrease in catalyst activity was observed, ranging from 45 TM/Kg to 44 TM/Kg. When the DMF concentration was increased to 40.23 ppm, the catalyst activity decreased to 43 TM/Kg, and with 75.32 ppm, it dropped even further to 39 TM/Kg. The highest concentration of DMF evaluated, 89.92 ppm, resulted in a catalyst productivity of 36.5 TM/Kg and lost productivity of 22%. In addition, significant changes in the polymer’s melt flow index (MFI) were noted as the DMF concentration increased. When 89.92 ppm of DMF was added, the MFI loss was 75%, indicating a higher flowability of the polymer. In this study, it was found that dimethylformamide (DMF) exhibits a strong affinity for the titanium center of a Ziegler–Natta (ZN) catalyst, with an adsorption energy (*E_ad_*) of approximately −46.157 kcal/mol, indicating a robust interaction. This affinity is significantly higher compared to propylene, which has an *E_ad_* of approximately −5.2 kcal/mol. The study also revealed that the energy gap between the highest occupied molecular orbital (HOMO) of DMF and the lowest unoccupied molecular orbital (SOMO) of the Ziegler–Natta (ZN) catalyst is energetically favorable, with a value of approximately 0.311 eV.

## 1. Introduction

Due to the significant development of coordination catalysis in the 1950s, the Ziegler–Natta heterogeneous catalysts based on titanium and magnesium were discovered, becoming critical elements in the olefin polymerization industry. These catalysts have been the subject of intense research due to their ability to selectively control the synthesis of polyolefins with highly desirable properties [1,2,3]. The Ziegler–Natta catalyst is recognized as one of the most critical catalysts in the industrial production of polyolefins, such as polyethylene and isotactic polypropylene. This catalyst system comprises four key elements that work together to facilitate polymerization in a controlled and selective manner. Firstly, the catalyst precursor, titanium chloride (TiCl_4_), acts as the active species that initiate the polymerization reaction. TiCl4 interacts with olefin monomers to form chemical bonds and give rise to the polymer chain. Secondly, a magnesium chloride (MgCl_2_) support is used, which stabilizes and provides a suitable structure for the catalyst. The TiCl_4_ adsorbs on the surface of the MgCl_2_, which gives it stability and facilitates its interaction with the other components. The third component is the electron donors (Lewis bases), molecules capable of interacting with the catalyst and influencing the stereoselectivity of polymerization. These donors modify the properties of TiCl_4_, allowing greater control over the structure and properties of the resulting polymer [4,5]. Catalyst activators, usually aluminum alkyl compounds, such as triethylaluminum (AlEt_3_), are the fourth component. The AlEt_3_ interacts with the TiCl_4_ adsorbed on the support, facilitating the polymerization reaction and promoting the formation of a highly active catalyst [6,7].

In recent years, exhaustive research has been carried out to develop improvements in the Ziegler–Natta catalyst system, exploring new variants of catalysts and the incorporation of additives and modifiers to optimize their performance. But, there are still challenges to overcome, such as the presence of poisons, which are substances that inhibit catalytic activity. Competition between multiple active sites affects the performance and efficiency of the ZN catalyst. These poisons can be by-products of the polymerization reaction, impurities in the reagents, or unwanted products. Its presence can decrease the catalytic activity and affect the selectivity and quality of the polymers obtained [8,9]. To better understand the poisoning of ZN catalysts during polypropylene synthesis, it is necessary to use theoretical and experimental tools [10,11,12]. The density functional theory (DFT) has established itself as an indispensable tool in investigating the inhibition of ZN catalysts. Through computational calculations, the DFT allows for analysis of the molecular interactions and the reaction mechanisms between the catalyst, the inhibitors, and the substrates, offering a precise understanding of the associated energy profiles. This theoretical approach provides crucial information to optimize the formulation of catalysts and design more efficient inhibitors, which improves the efficiency and selectivity in olefin polymerization [13,14,15,16]. There are computational studies using DFT where various ester compounds, including aromatic benzoate and silylester, were investigated. One of the most notable conclusions is that an excessive addition of these substances contributes to the poisoning of the catalyst’s active sites [17,18,19,20]. Additionally, the chemical reactions involved in the formation of these active sites have been studied using DFT [20,21,22,23,24].

In particular, a substance that can promote poisoning in polypropylene production is dimethylformamide (DMF), an organic chemical compound belonging to the amide family [25]. It is a colorless liquid with low volatility that is highly soluble in water and most organic compounds [18]. DMF is primarily used as an industrial solvent in various applications, including the production of polymers, copolymers, synthetic leather, polyacrylonitrile, polyurethane (Spandex) fibers, and pharmaceutical products. Besides its use as a solvent, DMF also possesses properties as a dehydrating agent and catalyst [19,20]. It is important to note that DMF, like most organic solvents, presents risks to health and the environment [21]. Various studies have shown that occupational exposure to DMF at concentrations below 30 mg/m^3^, which is the Threshold Limit Value (TLV) recommended by the American Conference of Governmental Industrial Hygienists (ACGIH), can intoxicate humans either by inhalation, ingestion, or absorption through the skin causing liver toxicity, the most common are hepatitis, fibrosis, cirrhosis, and even cancer [22]. In addition, studies reveal that DMF is a contaminant in industrial waters, which leads to an increase in nitrogen levels and the eutrophication of bodies of water, so adequate precautions must be taken during its handling and industrial use [23]. In a study conducted by Torabi et al. about the effects of DMF as the final electron donor in the synthesis of low-density polyethylene, it was concluded that at proportions more significant than the 0.25 DMF/Ti ratio, DMF begins to annihilate some of the active sites of the catalyst, especially those that are capable of producing linear chains. The increase in the said ratio reduces the activity of the catalyst, and there is a reduction in Mn and Mw and a slight increase in the average weight and number of short-chain branches.

Even though numerous studies have focused on the toxicity of DMF for humans and the environment, the influence of this solvent on the ZN catalyst during polypropylene synthesis has just been thoroughly investigated. Therefore, the objective of our research is to analyze the impact of traces of dimethylformamide (DMF) on the productivity of the Ziegler–Natta (ZN) catalyst using integrating computational and experimental methods. Through density functional theory (DFT) calculations and practical tests, we sought to understand how the presence of DMF affects the catalytic activity and selectivity of olefin polymerization. This research is innovative because it addresses a relevant challenge in the polymerization industry, where trace amounts of DMF can influence catalyst effectiveness and the quality of the resulting polymer. By combining theoretical and experimental approaches, we hope to provide valuable information for optimizing the formulation of ZN catalysts and designing more efficient inhibitors, improving yield and selectivity in polyolefin production.

## 2. Materials and Methods

### 2.1. Standards and Reagents

To carry out this work, a fourth-generation Ziegler–Natta catalyst supported on MgCl_2_ with a 3.6% titanium content in its composition was used. Additionally, diisobutyl phthalate (DIBP) was employed as a chemical donor, which was supplied by Sudchemie in Germany. To assist in the process, a cocatalyst known as triethylaluminum (TEAL), with 98% purity, was acquired from Merck in Darmstadt, Germany and was diluted in n-heptane. Furthermore, an external compound called cyclohexyl methyl dimethoxysilane (CMDS) was also obtained from Merck in Germany. The main raw material, isotactic polymeric grade propylene, was provided by Shazand Petrochemical in Tehran, Iran. The gases used, hydrogen and nitrogen, had a purity level of 99.999% provided by Lynde (St. Paul, MN, USA). Dimethylformamide was obtained from Sigma-Aldrich (St. Louis, MO, USA) with a purity of 99.99%.

#### Propylene Polymerization

In this research study, a pre-polymerization process based on the conventional procedure used by the chemical industries was carried out. The pre-polymerization of propylene was carried out in a 1 L round-bottom, jacketed, stainless-steel reactor equipped with a mechanical spiral stirrer and a circulating oil temperature control system. To start the process, the reactor was purged with nitrogen for 2 h at 70 °C and then cooled to 50 °C before transferring 210 mL of n-hexane to the reactor under a nitrogen atmosphere. Subsequently, 8 Kh/h of a cocatalyst, 5 Kh/h of a catalyst, and an appropriate amount of DMF was injected using a gas-tight syringe, all under a nitrogen atmosphere. At 70 °C, propylene was continuously supplied to the reactor at a constant flow rate for about 120 min, which resulted in obtaining a pre-polymer with a ratio of 40 g PP/mmol Ti. Once the pre-polymer was prepared, it was dried using a nitrogen flow at 60 °C. Then, to disperse the pre-polymer particles inside the reactor, the stirrer speed was increased to 1000 rpm. Gas phase polymerization was started, introducing the reagents into the same reactor used in the pre-polymerization stage. The gas phase polymerization was carried out under a total pressure of 70 bar at 70 °C for 120 min. It is relevant to mention that repeated experiments were carried out to verify the consistency of the results, as shown in Table 1.

### 2.2. Melt Flow Index (MFI) and Average Molecular Weight (Mw)

In this study, samples of virgin polypropylene (PP) with different standard melt flow values (MFI) were collected from various suppliers and the polymer processing industry. PP samples from the polymerization process were also obtained. All polymer samples were characterized using the MFI test method at a temperature of 230 °C and a load of 2.16 kg. The molecular weight distribution of PP was determined using the Branmer model, which has been used in other research and has shown excellent approximations [7,18,24].

### 2.3. Computational Methods

A conformational search was carried out to identify the stable structure of the synthesized compound with an empirical method using the Gaussian 16 program. The most stable structure obtained was optimized using the density functional theory (DFT) B3LYP (the hybrid functional of three parameters of Becke using the correlation functional of Lee, Yang, and Parr) [26] with the method 6-311 G(d,p). The energy values of the highest occupied molecular orbital (HOMO) and lowest unoccupied molecular orbital (LUMO) were calculated using density functional theory (DFT) at B3LYP/6-311 G(d,p) levels using the self-consistent reaction field (SCRF) approach.

We made adjustments to how the atoms were arranged in the molecules without symmetry constraints using the B3LYP method along with a 6-311G(d,p) basis set. We also took into account corrections to consider how electrons were dispersed among the atoms using the DFT-D3 method (zero damping). To ensure that the structures were well-optimized, we calculated how the molecules would vibrate at the atomic level.

In this study, we used a β-MgCl_2_(110) surface. The adsorption energy of the molecule on the surface is calculated using Equation (1):

(1) $$E_{ad}=E_{Mg/P}-E_{Mg}-E_{P}$$

where *E_Mg/P_* is the energy of the system composed of the adsorbed inhibitor on the catalyst, *E_Mg_* is the energy of the catalyst, and *E_P_* is the energy of the inhibitor.

We conducted a frequency analysis under standard conditions (1), atmosphere pressure, and 298 Kelvin temperature to calculate the enthalpy (*H_ad_*) and Gibbs free energy (*G_ad_*) of adsorption. This calculation is carried out using the same formula as found in Equation (1). The only distinction is that, instead of using zero-point energies, we employ the values of H and G in these calculations.

#### Molecular Electrostatic Potentials

In this study, optimized geometry calculations for the DMF molecule were carried out using the Gaussian 16 self-consistent field ab initio method, with the B3LYP base set known for its efficiency in determining molecular structures. Subsequently, the electron densities and electrostatic potentials were calculated using the 6-311G base set. The molecule was depicted surrounded by a three-dimensional surface showing the constant electron density contour. On this surface, the molecular electrostatic potentials were calculated and represented. These potentials can be visualized with different levels of detail, but our current qualitative purpose is to identify the most likely sites for nucleophilic, electrophilic, or free radical attack.

## 3. Results

### 3.1. Conditions and Study Variables

**Figure 1:**
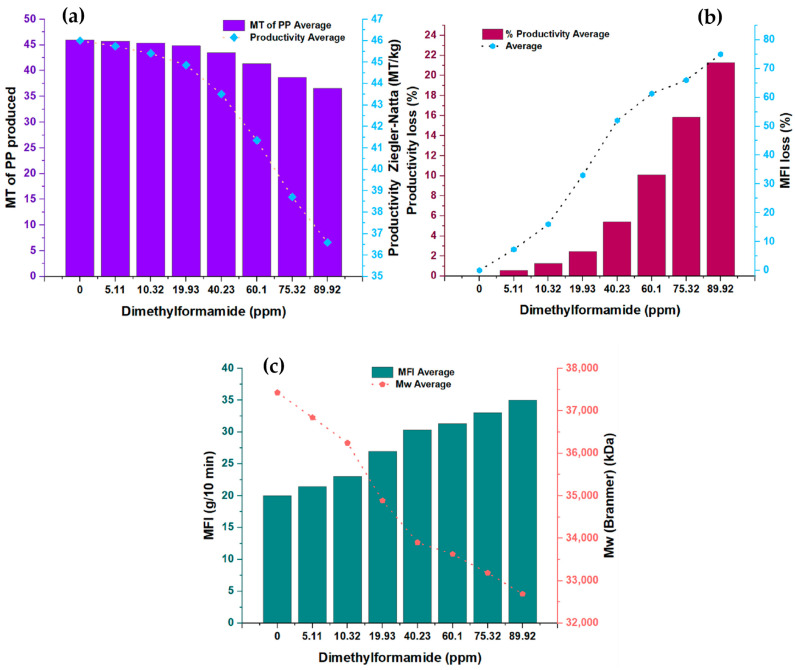
(**a**) Changes in the productivity and amount of TM produced in the presence of dimethylformamide; (**b**) modifications in the MFI of the PP produced and productivity losses in the presence of dimethylformamide; (**c**) behavior of MFI and Mw at different concentrations of dimethylformamide.

In the present study, Figure 1 shows the measurements made in pairs of variables concerning the concentration of N, N-dimethylformamide (DMF). Specifically, the relationship between the DMFA concentration and two key variables was analyzed: the amount of polypropylene (PP) produced, expressed in metric tons (MT), and the productivity of the Ziegler–Natta (ZN) catalyst, measured in terms of metric tons of PP per kilogram of solvent (MT/kg). In addition, two other variables were examined as a function of DMF concentration: percentage loss in catalyst productivity and melt flow rate. Likewise, the relationship between the melt flow index and the molecular weight of polypropylene was analyzed. Table 2 provides detailed information on the experimental conditions used and the amounts of the substances involved in this study. Just so you know, the conditions shown in Table 2 were the same for each run, which was 24 in total.

This analysis allowed us to explore how the DMF concentration affects multiple variables related to polypropylene production and the activity of the Ziegler–Natta catalyst. The results obtained offer a complete view of the effects of the DMF concentration in the process, which is relevant for the understanding and optimization of PP production systems in the chemical industry. The study also provides valuable information on the percentage loss in catalyst productivity, an essential measure for evaluating process performance. In addition, the physical properties of polypropylene, such as melt flow rate and molecular weight, were investigated to understand better how the DMF concentration can influence these fundamental characteristics of the final product.

#### 3.1.1. PP Productivity as a Function of the Inhibitor

Figure 1a presents the analysis of the relationship between the DMF concentration and the first two variables: the amount of PP produced in metric tons (MT) and the productivity of the ZN catalyst in metric tons of PP per kilogram of solvent (MT/kg). The synthesized PP had a density between 0.9009 g/cm^3^ and 0.9011 g/cm^3^.

A clear downward trend is observed in both variables as the DMF concentration increases, indicating an inversely proportional relationship. This graph illustrates how DMF affects the amount of PP produced and, in turn, directly impacts the productivity of the ZN catalyst. However, it is essential to highlight that this inhibitor shows a lower impact on catalyst productivity, precisely 23.93% less, compared to the furan inhibitor analyzed in a previous study [1]. These results highlight the importance of carefully considering the DMF concentration in PP production processes since its direct influence on the amount of PP generated and on the catalyst’s efficiency can significantly affect the overall productivity of the system.

#### 3.1.2. Loss in Productivity and Loss in Fluidity Depending on the Concentration of DMF

In Figure 1b, the analysis of the relationship between the DMF concentration and the second significant variable is presented: the percentage loss in catalyst productivity and the flow rate. A directly proportional relationship is observed between both variables as the concentration of DMF increases. The increase in the concentration of this inhibitor is correlated with a more significant loss in catalyst productivity, which directly impacts the length of the polymer chain formed. This loss in productivity results in changes in the melt flow index of the polymer. These results indicate that the DMF inhibitor influences the physical properties of the produced polymer.

#### 3.1.3. Melt Flow Rate and Molecular Weight as a Function of DMF Concentration

Figure 1c shows the analysis between the melt flow index (MFI) in grams per 10 min (g/10 min) and the molecular weight (Mw) in kilodaltons (kDa) of the DMF concentration. It is observed that the melt flow index increases as the DMF concentration increases while the molecular weight decreases. These findings conclusively confirm that the DMF inhibitor affects the properties of the polymer, precisely the length of the polymer chains generated. It is important to note that the melt flow index measures the polymer’s flowability under specific processing conditions. The increase in melt index indicates a higher flowability of the polymer, which may have implications for its behavior during manufacturing and subsequent applications. On the other hand, the decrease in the molecular weight of the polymer suggests that the polymer chains generated in the presence of DMF have a shorter length. Molecular weight is an important measure that determines the physical and mechanical properties of the polymer, such as its strength and durability. Therefore, the decrease in molecular weight indicates a significant alteration in the physical properties of the polymer generated in the presence of DMF. A notable point of interest in the results of Figure 1c is that the DMF concentration shows a directly proportional and almost systematic effect on the melt flow rate, except at the concentration point of 40.23 ppm, which may indicate a possible complex interaction between the DMF and the polymeric system at that specific concentration.

The decrease in the molecular weight and the increase in the melt index indicate a significant modification in the polymer structure generated using DMF. These results have important implications in the design and control of polypropylene production processes since the physical properties of the polymer are vital factors that determine its performance and applicability in various industries. In conclusion, Figure 1c shows clearly and precisely how the DMF concentration affects both the melt index and the molecular weight of the generated polymer. These results provide a solid scientific basis for understanding the effects of the DMF inhibitor on the physical properties of the polymer. They are relevant for optimizing polypropylene production processes in the chemical industry.

### 3.2. DMF Inhibition Mechanism

**Figure 2:**
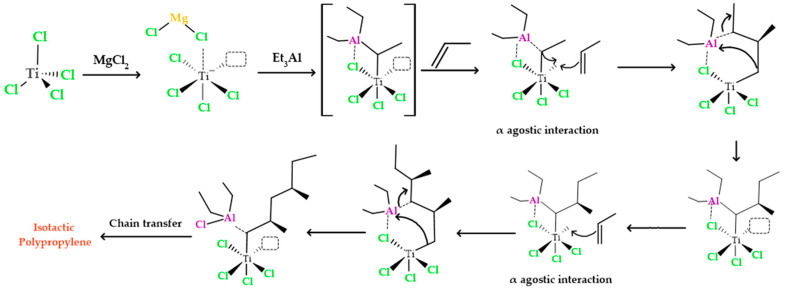
Polypropylene formation process with an isotactic structure using titanium tetrachloride and triethyl aluminum as catalyst and cocatalyst.

As illustrated in Figure 2, the initial step in the heterogeneous polymerization mechanism involves the formation of a complex between titanium and the cocatalyst. This generated complex between titanium and aluminum possesses an empty orbital, which interacts with the π electrons present in the monomer. This interaction leads to the formation of a π complex. The π complex, in turn, directs the -CH3 portion of the monomer away from titanium to aluminum. During this process, there is a rapid rearrangement of the π complex, resulting in the creation of bonds between the carbon atoms of propylene and titanium. At the same time, the second carbon atom in polypropylene gains a positive charge, while the aluminum alkyl group is transferred to the carbocation. This alkyl group transfer to the carbocation occurs instantly, without allowing the formed carbocation to reorganize. Therefore, the propylene group is stereospecifically inserted between titanium and aluminum through the action of the titanium complex. This titanium complex continues to insert additional propylene monomers between itself and the ethyl group that is ultimately added to the chain. As the reaction progresses, an isotactic polymer is formed.

**Figure 3:**
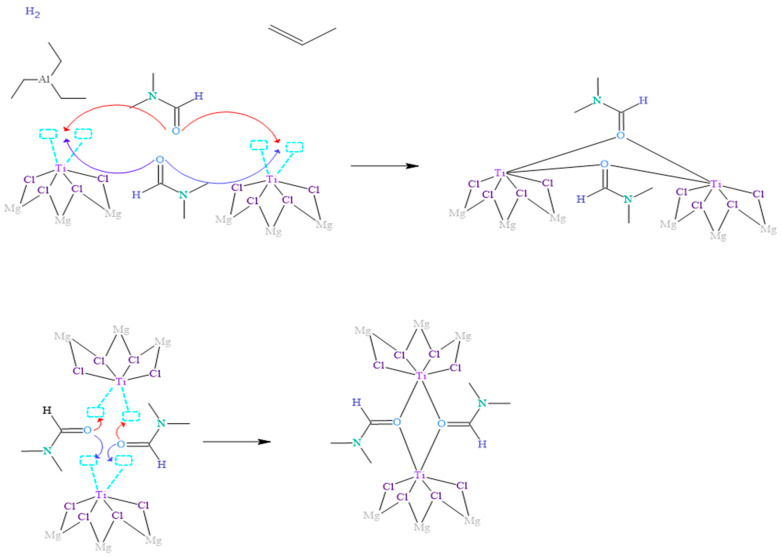
Proposed mechanism of inhibition of the Ziegler–Natta catalyst by the presence of DMF.

When inhibitors, such as N,N-dimethylformamide (DMF), are involved in addition to the reagents used in the isotactic polypropylene polymerization process, the essential reaction mechanism for polymerization is disrupted, as shown in Figure 3. Several studies have demonstrated that DMF coordinates with the metal centers through the oxygen atom, which has unpaired electrons with less steric hindrance.

In the context of the coordination complex formation, Figure 3 illustrates how two DMF molecules approach adjacent titanium metal centers and coordinate with them by forming oxo bridges. In this process, the oxygen atom shares the two available pairs of electrons, which co-occurs in both DMF molecules involved in the reaction. These DMF molecules compete and surpass other ligands, such as triethylaluminum and propylene, in their ability to react with metal centers. This is because the ligands capable of forming bridges in the coordination complexes, specifically oxygen in the oxo bridge, confer more excellent stability to the complexes formed due to more significant interaction of the orbitals involved in the bonds.

It is widely known that atoms in nature tend to prefer to bond in the way that implies the least energy under the conditions given at the moment of union; that is, they seek the most stable form. Therefore, it is justified that how DMF binds to the Ziegler–Natta (ZN) catalyst is shown in Figure 3. These findings provide a deeper understanding of the reaction mechanisms between DMF and metal centers, as well as the influence of these coordination complexes on the activity and selectivity of the ZN catalyst. The detailed knowledge of these processes allows the rational design and optimization of catalysts in producing polymers, such as polypropylene.

### 3.3. Analysis of the Use of DMF as an Inhibitor of the ZN Catalyst

In this study, we aimed to gain insight into how DMF impacts the ZN catalyst by employing density functional theory (DFT) at the B3LYP/6-311G(d,p) level. In this analysis, we assessed various quantum chemical parameters, such as the highest occupied molecular orbital energy (EHOMO), the lowest unoccupied molecular orbital energy (ELUMO), and the energy gap (ΔE). Additionally, we delved into local reactivity by utilizing Fukui indices to predict the locations where nucleophilic and electrophilic attacks may occur. The theoretical findings and correlations we have uncovered align well with the experimental results.

**Figure 4:**
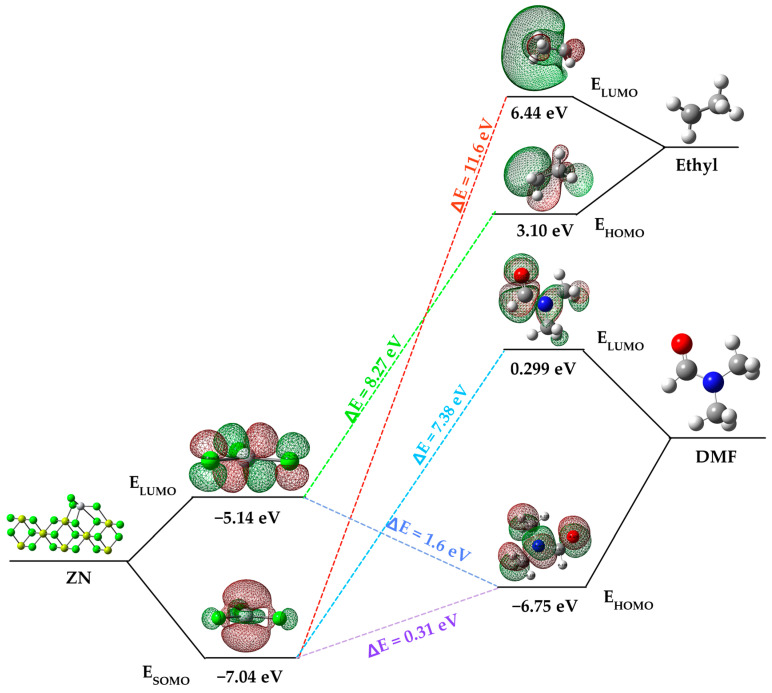
Frontier molecular orbital diagram of DMF, ethyl group and ZN catalyst.

The impact of an inhibitor compound’s ability to reduce activity is often attributed to how the molecule binds to the metal surface. This binding can manifest in two ways: physical (physisorption) or chemical (chemisorption), depending on the strength of this connection. In the case of chemisorption, one of the reactive molecules acts as an electron pair donor, while the other acts as an electron pair acceptor. The value of the highest occupied molecular orbital energy (EHOMO) is used to measure a molecule’s predisposition to donate electrons [27]. Higher EHOMO values indicate a greater tendency of the molecule to donate electrons to accepting molecules that have vacant and low-energy molecular orbitals. As EHOMO values increase, adsorption becomes easier, subsequently enhancing inhibition effectiveness by influencing the transport process through the adsorbed layer. On the other hand, ELUMO indicates the molecule’s capacity to accept electrons. In Figure 4, an energy diagram of the molecular orbitals of DMF and the ZN catalyst is presented and compared with the HOMO and LUMO of the ethyl group from the cocatalyst. This comparison is essential because, as indicated in Section 3.2, the initial step in the heterogeneous polymerization mechanism involves the formation of a complex between titanium and the cocatalyst.

Focusing on the ZN catalyst and dimethylformamide (DMF), a significant difference in the energy values of their molecular orbitals is evident. DMF’s highest occupied molecular orbital (HOMO) has a higher energy level (−6.75 eV) compared to the ZN catalyst’s singly occupied molecular orbital (SOMO) (−7.04 eV). Similarly, DMF’s lowest unoccupied molecular orbital (LUMO) is higher in energy (0.01139) compared to the ZN catalyst’s LUMO (−0.18998). This indicates that DMF has a greater predisposition to donate electrons to accepting molecules, in this case, to the active titanium center of the ZN catalyst, which possesses a vacant molecular orbital with low energy, resulting in chemisorption between dimethylformamide and the catalyst’s active center.

The energy gap (ΔE = ELUMO − EHOMO) is an important parameter related to the inhibitory molecule’s reactivity toward adsorption on the metal surface. As ΔE decreases, the molecule’s reactivity increases, leading to an increase in the inhibitory efficiency (%IE) of the molecule. Lower values of the energy difference will provide good inhibition efficiency because the energy required to remove an electron from the last occupied orbital will be low [28]. It is relevant to note that the energy gap between the catalyst’s SOMO and DMF’s HOMO is energetically more favorable (ΔE = 0.311 eV) compared to other interactions, such as that between the catalyst’s SOMO and the LUMO of the ethyl group (ΔE = 11.6 eV) originating from the cocatalyst (AlEt_3_), and the SOMO of the catalyst and the HOMO of the ethyl group (8.27 eV).

#### 3.3.1. Molecular Electrostatic Potential Map

The molecular electrostatic potential (MEP) is a valuable tool used to investigate global molecular structure and reactivity, since it provides information about the charge distribution and the availability of electrons in a molecule. Using colors, the MEP indicates the areas with the highest electron density (red) or electron deficiency (blue) in the molecule. This allows the identification of nucleophilic sites, where the molecule has a higher probability of donating electrons (red areas), and electrophilic sites, where it has a higher affinity for accepting electrons (blue areas) [29].

**Figure 5:**
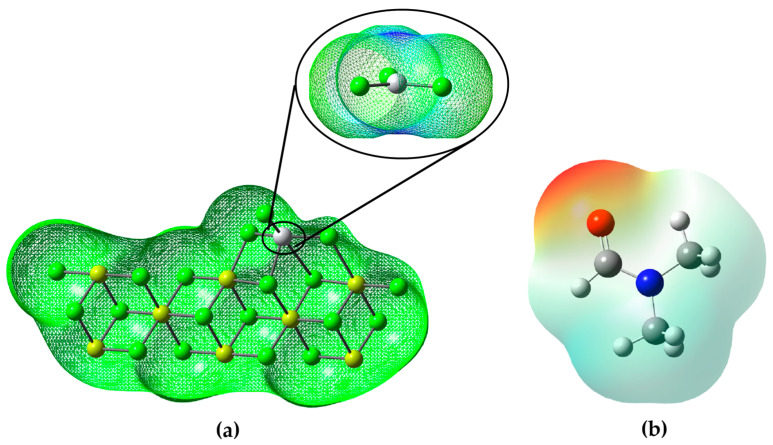
(**a**) Electrostatic potential map of the Ziegler–Natta catalyst; (**b**) electrostatic potential map of dimethylformamide.

According to the analysis of the molecular electrostatic potential (MEP) of the DMF (Figure 5b), it can be observed that the molecule presents different regions with different electron densities. The red highlighted areas in the MEP indicate areas where DMF has a higher electron density and affinity to accept electrons. These red areas correspond to the electrophilic sites of the molecule. On the other hand, the areas highlighted in blue in the MEP indicate regions with electron deficiency and, therefore, present a greater probability of donating electrons. These blue areas correspond to the nucleophilic sites of the molecule.

In the context of the inhibition of the ZN catalyst, this information from the DMF MEP is relevant because it suggests that DMF may act as an inhibitor by interacting with electrophilic sites on the catalyst. Since the catalyst ZN is probably a nucleophilic agent, DMF, with its electrophilic regions, could attract and form a stable complex with the catalyst, thus preventing the catalyst from reacting with the desired reagent and thus inhibiting its catalytic activity (look at the Figure 5b).

It is evident that the main region of the Ziegler–Natta catalyst is highlighted by its blue hue, located above the titanium atom. This blue coloration (Figure 5a) indicates an electron deficiency in that area and, consequently, a greater propensity to accept electrons.

In essence, DMF could function as a ZN catalyst inhibitor by competing for interaction with the catalyst at their electrophilic sites, leaving fewer sites available for the catalyst to bind to the reactant (either AlEt_3_ or propylene or whatever) and carry out its catalytic reaction. This interaction of the DMF with the ZN at their electrophilic sites would be similar to how enzyme inhibitors bind to the active sites of enzymes, preventing their activity.

#### 3.3.2. Fukui Functions

**Figure 6:**
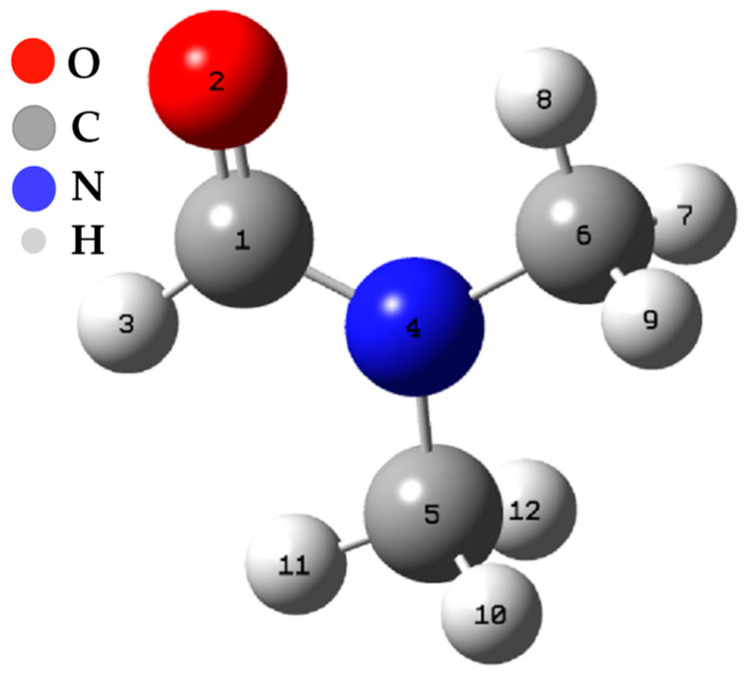
Spatial conformation of the DFM.

The calculations of the Fukui functions were carried out using the Hirschfeld population method [30]. The results of these calculations have been compiled and presented in Table 3. These calculations are centered on all the atoms present in dimethylformamide (see Figure 6).

An analysis of the values of the Fukui functions and the dual descriptor reveals the distribution of electron density in the DFM molecule and which atoms are more prone to participate in chemical reactions. For example, positive values of ${“f}^{-}”\;$ suggest that the corresponding atoms have a more remarkable ability to accept electrons, making them potential nucleophilic sites. On the other hand, positive “$f^{+}$” values indicate that atoms are more likely to donate electrons, making them electrophilic sites. Likewise, the negative value of “∆f” for some atoms indicates that they can act as centers of a negative charge, while positive values indicate centers of a positive charge. On the subject of ZN catalyst inhibition, this information is relevant, as it can help to understand how DMF interacts with the catalyst and how it affects the reactivity of the atoms in the DFM molecule. For example, if DMF has a high electron density at certain atoms, it could compete with the cocatalyst for binding to the catalyst and thus inhibit its catalytic activity. Furthermore, the dual descriptor (∆f) can indicate which atoms have a higher probability of changing their electronic charge during the interaction with the catalyst, which can also affect their ability to react.

The N3 and O12 carbon atoms stand out as the sites most prone to an electrophilic attack, meaning they have a high capacity to accept additional electrons. On the other hand, C1 and O12 atoms are the most susceptible to a nucleophilic attack, indicating their predisposition to donate electrons. In addition, the C1, N3, and O12 atoms are the most susceptible sites to an attack by free radicals, suggesting they can participate in electron transfer reactions. Table N + 1 shows the variation of ∆f depending on the atoms, and these results offer a more detailed view of the molecule’s reactivity. The nitrogen atom shows the most negative value of ∆f (−0.5702), making it the site most prone to an electrophilic attack. On the other hand, the C1 and O12 atoms present positive values of ∆f (0.4828 and 0.1324, respectively), which identifies them as the most favorable sites for a nucleophilic attack.

### 3.4. Interaction between Dimethylformamide and the Titanium Active Center

**Figure 7:**
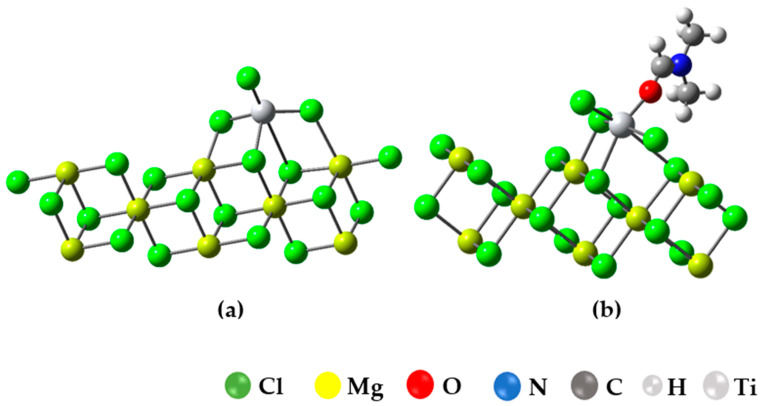
(**a**) Optimized structure of the Ziegler–Natta catalyst (TiCl_3_-Mg_8_Cl_16_); (**b**) surface (100) optimized structure for the union of DMF in Ti in the Mg_8_Cl_16_-TiCl_3_ model.

In this section, we present the results of simulating the binding of the inhibitor to the active TiCl_3_/Mg_8_Cl_16_ center, as illustrated in Figure 7a. The choice of this model is based on previous calculations that suggest that the coordination of TiCl_4_ with the (104) plane is quite weak or even unstable, whereas the coordination of TiCl_4_ with the (110) plane is energetically favored [8,9,10,11,12,13,14,15,16,17,18,19,20,21,22,23,24,25,26,27,28,29,30].

In the context of this study, we assessed the adsorption of dimethylformamide (DMF) on the titanium center of a Ziegler–Natta (ZN) catalyst and compared it to the value reported in the study [8], where the adsorption energy for propene on the titanium active center was reported. The results revealed that DMF exhibits a strong affinity for the titanium center, with an adsorption energy (*E_ad_*) of −46.157 kcal/mol, indicating a robust interaction. On the other hand, propylene showed an *E_ad_* of −5.2 kcal/mol, suggesting a lower affinity compared to DMF. This significant difference in adsorption affinity has important implications for chemical and adsorption processes, highlighting DMF’s ability to form strong bonds with the ZN catalyst, which can influence its behavior in chemical reactions and catalytic processes.

As a result, this “poison” hinders the formation of complexes with alkenes and alkene insertion reactions. It is important to note that this type of inhibition is virtually reversible: When the poison is removed from the system, the active centers resume the polymerization reaction. Furthermore, we calculated the Δ*G_ad_* of the complex formed between DMF and the ZN catalyst, obtaining a value of −30.6 kcal/mol, along with a Δ*H_ad_* of −47.6 kcal/mol. This indicates that this interaction is more favorable compared to other inhibitors, such as CH_3_OH, which has *E_ad_* and Δ*H_ad_* values of −29.1 and −26.5 kcal/mol, respectively, according to the same previously mentioned study.

## 4. Conclusions

In this study, we explored how the concentration of N, N-dimethylformamide (DMF) affects various variables related to polypropylene (PP) production and the activity of the Ziegler–Natta (ZN) catalyst. We analyzed the relationships between the DMF concentration and the amount of PP produced, as well as the ZN catalyst productivity measured in metric tons of PP per kilogram of solvent (TM/kg). We also examined other variables, such as the percentage loss in catalyst productivity and the polymer melt index. The results revealed a clear trend of decreasing PP production and ZN catalyst productivity as the DMF concentration increased, suggesting an inversely proportional relationship between these variables and DMF concentration. Additionally, a directly proportional relationship was observed between DMF concentration and the percentage loss in catalyst productivity and the polymer melt index. Regarding the physical properties of polypropylene, it was found that the melt index increased, and the molecular weight decreased as the DMF concentration increased. These findings indicate a significant modification in the polymer’s structure induced by DMF, which could impact its behavior during manufacturing and subsequent applications. It was also observed that dimethylformamide (DMF) has a strong affinity for the titanium center of a Ziegler–Natta (ZN) catalyst, with an adsorption energy (*E_ad_*) of approximately −46.157 kcal/mol. This affinity is significantly higher compared to propylene, which has an *E_ad_* of approximately −5.2 kcal/mol. Furthermore, the study revealed that the energy gap between the highest occupied molecular orbital (HOMO) of DMF and the lowest occupied molecular orbital (SOMO) of the Ziegler–Natta (ZN) catalyst is energetically favorable, with a value of approximately 0.311 eV. Additionally, the analysis of the molecular electrostatic potential (MEP) revealed that DMF has electrophilic regions, indicating its potential interaction with the electrophilic sites of the catalyst, acting as an inhibitor.

## Figures and Tables

**Table 1:** Collection of PP samples with traces of dimethylformamide.

Rehearsal	Replica	Dimethylformamide (ppm)	Productivity ZN (MT/Kg)	% Lost Productivity
1	1	0	46	0
1	2	0	46	0
1	3	0	46	0
2	1	0.35	45.81	0.56
2	2	0.34	45.77	0.5
2	3	0.36	45.72	0.61
3	1	0.75	45.56	1.26
3	2	0.74	45.39	1.37
3	3	0.73	45.46	1.17
4	1	1.55	44.82	2.43
4	2	1.54	44.81	2.52
4	3	1.51	44.85	2.46
5	1	3.1	43.49	5.41
5	2	3.2	43.48	5.35
5	3	3.1	43.46	5.46
6	1	5.1	41.19	9.96
6	2	5.2	41.17	10.06
6	3	5.1	41.15	10.28
7	1	7.5	38.55	15.93
7	2	7.6	38.47	15.78
7	3	7.4	38.53	15.85
9	1	10.1	36.53	21.3
9	2	10.05	36.45	21.3
9	3	10.2	36.39	21.3

**Table 2:** Materials and conditions of each of the components of the PP polymerization process.

Materials and Conditions	Value	Unit
Catalyst	5	Kg/h
Propylene	1.2	TM/h
TEAl	0.25	Kg/h
Hydrogen	30	g/h
Selectivity control agent	1	mol/h
Temperature	70	°C
Pressure	27	bar

**Table 3:** Local descriptors of dimethylformamide.

Number	$f^{-}$	$f^{+}$	$f^{0}$	∆f
1	0.0171	0.4999	0.2585	0.4828
2	0.0005	0.0012	0.0008	0.0007
3	0.6043	0.0341	0.3192	−0.5702
4	0.0200	0.0256	0.0228	0.0056
5	0.0069	0.0016	0.0042	−0.0053
6	0.0048	0.0054	0.0051	0.0006
7	0.0640	0.0019	0.0330	−0.0622
8	0.0129	0.0972	0.0551	0.0843
9	0.0050	0.0007	0.0029	−0.0043
10	0.0154	0.0254	0.0204	0.0100
11	0.0756	0.0014	0.0385	−0.0743
12	0.1733	0.3057	0.2395	0.1324

## Data Availability

Not applicable.
